# Using Multicriteria Decision Analysis to Support Research Priority Setting in Biomedical Translational Research Projects

**DOI:** 10.1155/2015/191809

**Published:** 2015-10-01

**Authors:** Gimon de Graaf, Douwe Postmus, Erik Buskens

**Affiliations:** Department of Epidemiology, University of Groningen, University Medical Center Groningen, 9700 RB Groningen, Netherlands

## Abstract

Translational research is conducted to achieve a predefined set of economic or societal goals. As a result, investment decisions on where available resources have the highest potential in achieving these goals have to be made. In this paper, we first describe how multicriteria decision analysis can assist in defining the decision context and in ensuring that all relevant aspects of the decision problem are incorporated in the decision making process. We then present the results of a case study to support priority setting in a translational research consortium aimed at reducing the burden of disease of type 2 diabetes. During problem structuring, we identified four research alternatives (primary, secondary, tertiary microvascular, and tertiary macrovascular prevention) and a set of six decision criteria. Scoring of these alternatives against the criteria was done using a combination of expert judgement and previously published data. Lastly, decision analysis was performed using stochastic multicriteria acceptability analysis, which allows for the combined use of numerical and ordinal data. We found that the development of novel techniques applied in secondary prevention would be a poor investment of research funds. The ranking of the remaining alternatives was however strongly dependent on the decision maker's preferences for certain criteria.

## 1. Introduction

The difficulty of developing biomedical discoveries into new medical technologies or therapies has been widely recognized and is often referred to as the “bench-bed gap” or the “valley of death” [1, 2]. Translational research aims to bridge this gap by integrating the societal needs identified at the bedside with the research done at the bench. It encompasses the entire value chain from basic biomedical research, through epidemiology, clinical testing, product development, policy and regulatory compliance, and marketing. As a result, the overall success of a translational research project is determined by a multitude of technological, clinical, economic, and regulatory factors. All these factors need to be considered when evaluating which of the available research strategies are most likely to yield innovations that will eventually gain widespread adoption in daily clinical practice. This makes priority setting for translational research a complex problem that requires decision makers to gather and synthesize expertise from different fields. Without the use of a formal decision support method, it is generally impossible to simultaneously consider all aspects of such a decision problem, making it likely that too much emphasis is put on a single outcome of the translational research process. In such a setting, the use of multicriteria decision analysis (MCDA) can assist in structuring the problem and in making the decisions justifiable and replicable, thereby increasing accountability for public resources spend [3].

In the context of government-sponsored technology development programs, MCDA has previously been applied to support the selection of research and development projects across different industries and focus areas [4, 5]. However, these applications are not directly portable to research priority setting in biomedical translational research projects as the healthcare industry has specific properties that were not addressed in these studies. In particular, healthcare markets are heavily regulated and public provision of goods and services plays an important role in these markets. These characteristics impose rather strict constraints with respect to market penetration and price setting that already need to be considered early during the translational research process. In this paper, we demonstrate how these aspects can be incorporated in a formal way by using MCDA for priority setting at the start of a translational research project. We illustrate this by means of a case study conducted within the context of a translational research project aimed at the prevention of type 2 Diabetes Mellitus (DM2) and its related complications.

## 2. Application of MCDA to Research Priority Setting in Biomedical Translational Research Projects

**Figure 1:**
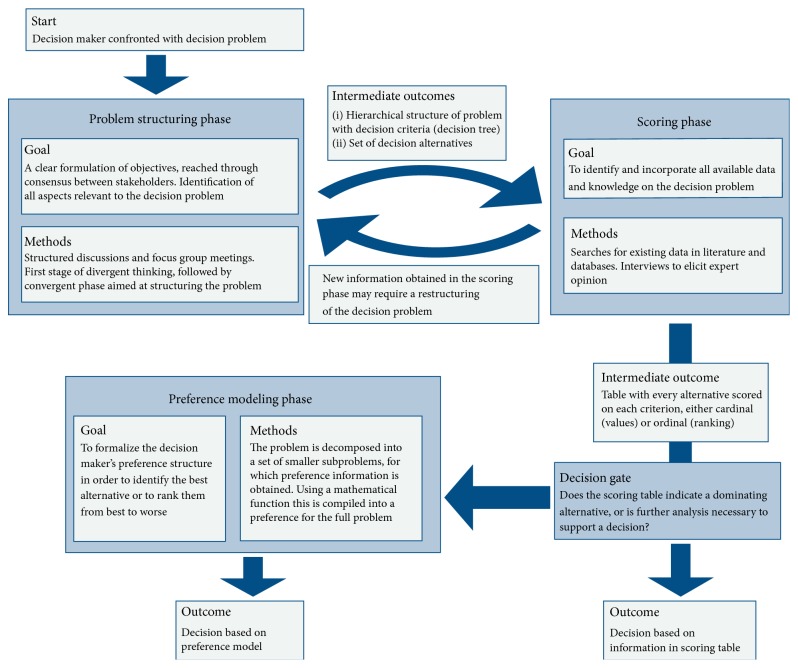
Schematic overview of the application of multicriteria decision analysis for priority setting.

Research priority setting for biomedical translational research is a complex problem that requires decision makers to consider a multitude of technological, clinical, economic, and regulatory factors. In such situations, the use of a formal decision support method encourages the incorporation of views and knowledge from experts in different parts of the value chain of biomedical research, thereby reducing the possibility that at later stages in the product development process problems are encountered that in hindsight could already have been foreseen at the start of the project. It can also ensure that all available information related to the decision problem is incorporated into the decision making process, thereby reducing the chance that the decision focuses too much on a single or narrow set of aspects of the problem. Within the framework of MCDA, this is achieved by sequentially going through the following three phases: problem structuring, scoring of the alternatives against the criteria, and preference modeling (Figure 1). Each of these phases is briefly described in the subsections below.

### 2.1. Problem Structuring

During problem structuring, the different stakeholders involved in the decision making process express their knowledge and views on the context of the decision problem as well as their objectives regarding the decision. Several formats and tools have been proposed to support this idea generation process, including “Post-It” sessions and various checklists and other aids to thinking such as adopting different perspectives and identifying barriers and constraints [3]. This divergent mode of thinking is followed by a convergent phase of idea structuring, in which ideas are clustered and aggregated to arrive at a set of decision alternatives (if not yet clearly defined at the start of the process) and a set of criteria against which these alternatives are to be evaluated. Depending on the decision context, the definition of these criteria can to an extent be informed by objective knowledge of relevant cause-and-effect mechanisms from scientific literature or other sources. However, the criteria should reflect the objectives of the relevant decision makers and therefore should be derived from discussions with the decision makers. Knowledge from outside the decision maker group can be incorporated into these discussions but should never dictate criteria by itself. The output of the problem structuring phase is often a value tree. This is a graphical representation of the hierarchical ordering of the criteria.

### 2.2. Scoring of the Alternatives against the Criteria

The next step is to score the alternatives against these criteria, which is done at the lowest level of the value tree. For some criteria (e.g., cost), it may be possible to assess the performance of the alternatives numerically, whereas, for others (e.g., quality), it may only be feasible to obtain an ordinal ranking of the alternatives or to allocate them to verbally defined levels of performance (e.g., poor, reasonable, and excellent). How the alternatives are scored against the criteria differs from decision context to decision context and depends, amongst others, on the amount of data (e.g., results from observational and/or experimental studies, output from mathematical models, or expert opinion) that is available at the start of the decision making process and on how many resources one is willing to invest in the collection of more precise measurements. As the information obtained in the scoring phase can change the perspective on the decision problem, it might be necessary to revert to the problem structuring phase in order to incorporate these new insights in the decision context. If this is not the case, the end of the scoring phase concludes the formal specification of the decision problem.

Based on the information in the scoring table, it is sometimes possible to identify one or more alternatives for which there is at least one other alternative that performs better on all of the criteria included in the decision problem. As it is never optimal to select one of these dominated strategies, they can safely be eliminated from the set of decision alternatives. If there is sufficient budget to fund all the remaining strategies, the decision problem is solved, meaning that the multicriteria decision making process can be ended after the scoring phase. If not, the set of decision alternatives needs to be further reduced by making value trade-offs among the performance levels on the different criteria. In such situations, the use of preference modeling can assist in formalizing the decision makers' preference structures, thereby reducing the chance that the decision focuses too much on a single aspect of the decision problem.

### 2.3. Preference Modeling

At the research priority setting stage of a translational research project, the amount of developmental uncertainty surrounding the conceived product concepts is usually still enormous. As a result, a full quantitative assessment of the expected clinical and economic benefits from each of the identified decision alternatives is generally not yet possible. It is therefore likely that for some of the criteria the data in the scoring table are solely based on expert opinion. As experts are often more comfortable with producing rankings (e.g., the number of competitor products is larger for alternative A than for alternative B) than with providing exact numerical estimates (e.g., there are 10 competitor products for alternative A and 6 for alternative B), it is important that such ordinal data can be accommodated in the preference modeling phase. For this reason, we will focus in this section on describing SMAA-O [6], a variant of the stochastic multicriteria acceptability analysis (SMAA) method [7, 8] that has been developed for decision problems where the data for some or all criteria is ordinal.

In SMAA-O, it is assumed that the decision maker's preference structure can be represented by means of a mathematical function *v*(*x*) that is constructed in such a way that alternative *i* is preferred over alternative *j* if and only if *v*(*x* ^*i*^) > *v*(*x* ^*j*^), where *x* ^*i*^ denotes the column of the scoring table associated with alternative *i*. To simplify the construction of *v*(*x*), it is generally assumed that the criteria satisfy the independence conditions for applying the additive value function *v*(*x*, *w*) = *w*_1_ *v*_1_(*x*_1_) + ⋯+*w*_*n*_ *v*_*n*_(*x*_*n*_), where *n* is the number of criteria and *w*_*k*_ is the weight attached to criterion *k*. The partial value functions *v*_*k*_(*x*_*k*_), normalized so that the worst possible score on each criterion is assigned a value of 0 and the best possible score is assigned a value of 1, reflect the relative desirability of the different levels of achievement on the individual criteria. For numerical criteria, it is usually assumed that equal size ranges on the measurement scales represent the same amount of value to the decision maker, resulting in partial value functions that are linear. For ordinal criteria, the use of such a linear mapping between scale values and partial values is however not directly suitable as the distance between ranks on an ordinal scale is not known. In SMAA-O, this problem is dealt with by randomly assigning the scale values on the ordinal scale to partial values between 0 and 1, in such a way that the rank order between the scale values is maintained. Different ordinal to partial value mappings may translate into a different ranking of the decision alternatives as the overall value associated with each of these alternatives may change. This uncertainty is captured by the* rank acceptability indices b*_*i*_^*r*^, which describe the fraction of Monte Carlo iterations for which alternative *i* is ranked at place *r*. The* pairwise winning indices c*_*ij*_ describe the fraction of Monte Carlo iterations for which alternative *i* is ranked at a higher place than alternative *j*. Missing or imprecise information with respect to the values of the weights can be handled in a similar way by sampling the weight vector from a uniform distribution in the feasible weight space induced by the available preference information.

## 3. Case Study

### 3.1. Decision Problem

The prediction and early diagnosis of diabetes and diabetes-related cardiovascular complications (PREDICCt) project of the Center for Translational Molecular Medicine (CTMM) was initiated to enhance the possibilities for prevention of DM2 and associated complications through the development of methodologies for molecular diagnostics and molecular imaging of novel biomarkers associated with the development of DM2 and its related complications. DM2 is a complex disease with many genetic, environmental, and behavioral determinants as well as biological pathways involved. Additionally, it is a chronic disease that takes a long time to develop. As a result, there are many different possible target applications for novel diagnostic and imaging techniques. Not all target applications are however equally likely to achieve the objectives of the project to the same extent. As a result, a decision had to be made on the priority setting for the investment of available resources.

### 3.2. Problem Structuring

#### 3.2.1. Methods

Several discussion sessions were held with various researchers from the PREDICCt project. During these discussions multiple perspectives on the decision problem were suggested by participants and discussed in the group. Based on these discussions, a set of alternatives was defined. The business plan of CTMM, in which the stakeholders in the project expressed their views and interests, served as the starting point to define a set of criteria. All statements concerning objectives were isolated from the business plan and subsequently ordered and grouped.

#### 3.2.2. Results

As the main aim of the PREDICCt project was the prevention of DM2 and associated complications, the decision alternatives were defined in the scope of the preventive medicine framework. Preventive medicine is often classified in three different levels. Primary prevention targets those in whom the disease is not yet present, with the aim to provide interventions to prevent the disease from manifesting. Secondary prevention targets those who have the disease but are not yet symptomatic, aiming to reduce the morbidity through early treatment. Tertiary prevention is aimed at those who are diagnosed with the disease and enables the provision of interventions limiting further morbidity caused by complications. Complications of DM2 are an important aspect in this case, as most of the burden of the disease is caused by these complications [9]. There are two distinct categories of complications: microvascular (diabetic nephropathy, neuropathy, and retinopathy) and macrovascular (coronary artery disease, peripheral arterial disease, and stroke) [10]. These two categories of complications have distinct approaches to prevention, diagnosis, and care. Therefore, it was considered important to make a distinction between tertiary prevention aimed at microvascular complications and tertiary prevention aimed at macrovascular complications. The 4 alternative research approaches identified for the development of a novel biomarker technology in DM2 were thus as follows:

A biomarker technology applied to the general population to

1. select individuals eligible for interventions aimed at preventing or delaying the onset of DM2 (primary prevention),
2. identify those with undiagnosed diabetes in order to initiate treatment earlier (secondary prevention).

A biomarker technology applied to the population of diagnosed DM2 patients to

(3) select those that would benefit from interventions aimed at preventing or delaying* microvascular* complications (tertiary prevention),
(4) select those that would benefit from interventions aimed at preventing or delaying* macrovascular* complications (tertiary prevention).

The structuring of objectives from the business plan resulted in the identification of four main objectives: reduce the burden of disease, reduce healthcare costs, increase economic activity, and obtain a high academic profile.

**Figure 2:**
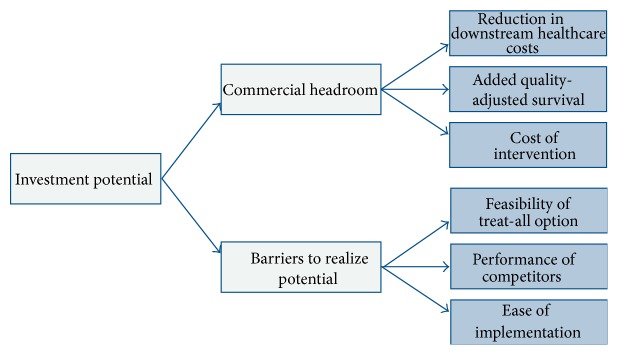
Value tree of overall and lower-level objectives of the public-private partnership.

The profile of academic output is to a large extent determined by the novelty and quality of scientific work presented. This is not directly related to the decision alternatives at hand, meaning that a high academic profile could be obtained no matter what alternative is chosen. This objective was therefore not considered relevant for the purpose of the present analysis. For the other three objectives, we conducted a literature review and a brainstorming session to identify a set of factors that are important determinants of these objectives and to identify potential barriers and constraints that hinder their achievement. This resulted in the value tree depicted in Figure 2.

In the healthcare technology market, the commercial potential of a product is dependent on its clinical value and its impact on the downstream healthcare consumption. The extent of this relation is determined by the level of regulation, which differs between jurisdictions as well as between different parts of the healthcare system. For highly regulated parts of the healthcare system, the impact of these factors on a technology's commercial potential can be assessed quantitatively by conducting a headroom analysis [11]. The rationale behind this approach is that the estimated change in health effects and healthcare costs, both direct and indirect, resulting from the implementation of a new technology determine the value of the technology for society, and thereby the maximum device-related cost which the use of this new product will still be reimbursed. As this cost provides an upper-bound for the price that the producer can charge for its product (the principle of value-based pricing), the amount of headroom available is a suitable proxy for the commercial potential of a new medical technology. The upper arm of the value tree therefore consisted of the following 3 determinants of the commercial headroom available: the decrease in downstream healthcare cost, the increase in quality-adjusted survival, and the cost of the intervention associated with the diagnostic or prognostic test. The 3 criteria forming the lower arm of the value tree captured the likelihood that the availability of a more accurate diagnostic or prognostic test will trigger changes in how the healthcare system currently operates. The feasibility of a treat-all option indicated the added value of the ability to treat specific patients as opposed to treating all patients. This provided an indication of the value stemming from better discrimination or prediction. Furthermore, the existence of high-quality competitor technologies, or lack thereof, was considered a major driver for the success of a novel technology to gain market share. Lastly, not all decision alternatives were considered equal in terms of the accessibility of the market and the ease of implementation in the clinical protocol. Technologies that readily fit within the practice as outlined by current guidelines can be implemented with relative ease. Contrarily, those that require a major change in clinical or public health protocols, for example, the initiation of a universal screening program, cannot fulfill their potential until such changes are established.

### 3.3. Scoring of the Alternatives against the Criteria

#### 3.3.1. Methods

For each of the decision alternatives, quantitative estimates of the decrease in downstream healthcare costs, the increase in quality-adjusted survival, and the intervention costs were available in the literature. The performance of the decision alternatives on these criteria was therefore expressed numerically. The performance on the other 3 criteria is strongly dependent on the type of technology developed and can therefore not be quantified at this stage. We therefore used expert opinion to formulate an ordinal ranking of the decision alternatives with respect to these criteria.

#### 3.3.2. Results

The complete scoring matrix is shown in Table 1. Estimates of the effects of primary prevention of diabetes and tertiary prevention of macrovascular complications on the reduction of downstream healthcare costs, gain of quality-adjusted survival, and the costs of interventions were based on a modeling study [12]. For the primary prevention scenario, a lifestyle intervention program in obese individuals was modeled, and for the tertiary prevention of macrovascular complications, a multifactorial treatment scenario combining intensive glycemic control, cholesterol-lowering treatment, and antihypertensive treatment was modeled. Estimates of the reduction in downstream healthcare cost, gain of quality-adjusted survival, and the costs of interventions for tertiary prevention of microvascular complications were based on a study that modeled the results of intensive blood glucose control and use of ACE-inhibitors on nephropathic complications [13]. As studies have found that secondary prevention of DM2 has little to no effect on downstream healthcare costs and quality-adjusted survival, the performance of this alternative on these two criteria was set equal to 0 [14]. However, in case screening is performed and patients are discovered, they will be treated. Therefore, the treatment costs of diabetes patients without complications were included [15].

Two main aspects contributed to the ranking of the feasibility to treat-all criterion: the budget impact and lack of implementation of existing cost-saving interventions. Primary and secondary prevention were ranked as highest and second highest as providing treatment to all individuals eligible for screening would not be feasible due to budget impact reasons. Within tertiary prevention, the microvascular complication alternative was ranked lowest as cost-saving interventions are readily available there but not yet fully implemented [13]. The barriers to implement such interventions must therefore first be overcome before the improved risk stratification possibilities can be implemented. Considering the performance of existing competing technologies, secondary prevention was ranked lowest. There, the diagnosis of diabetes itself cannot be improved as the disease is defined on measurements with the gold standard (glucose measurements). Additionally, there are numerous prescreening tools available that perform well and cost little (risk questionnaires) [16]. As a result of the latter, primary prevention was ranked second lowest. On the contrary, such risk stratification tools are hardly available and perform less well, for microvascular complications and to a lesser extent macrovascular complications. Lastly, the primary and secondary prevention settings of diabetes would necessitate some form of screening. Such a public health program could take years before being realized. This entails a serious problem for the implementation of any biomarker technology. As diagnosed diabetes patients regularly consult a physician, access to the patient is less problematic in the case of tertiary prevention.

### 3.4. Preference Modeling

#### 3.4.1. Methods

The partial value functions for the numerical criteria were obtained by linearly rescaling the criteria measurements to the interval [0,1], with the values of 0 and 1 assigned to the worst and best levels of performance on these criteria, respectively. The rankings of the alternatives on the ordinal criteria were randomly mapped to partial values between 0 and 1 consistent with these rankings by using the SMAA-O method. With respect to the weights, we specified three scenarios. First, we considered a base case scenario in which no additional constraints on the values of the weights were incorporated. The results of such a preference-free analysis can be used to eliminate alternatives that always fall short to at least one other alternative, irrespective of the decision maker's preferences. Second, we considered a scenario where a large commercial headroom was considered more important than avoiding barriers to realize potential, implying that *w*_1_ + *w*_2_ + *w*_3_ > *w*_4_ + *w*_5_ + *w*_6_. Lastly, we considered a scenario where the previous preference statement was reverted, implying that *w*_1_ + *w*_2_ + *w*_3_ < *w*_4_ + *w*_5_ + *w*_6_. All analyses were conducted in R (version 3.0.1) using the smaa (version 0.1.1) and hitandrun (version 0.2.2) packages that are available from CRAN.

#### 3.4.2. Results

**Figure 3:**
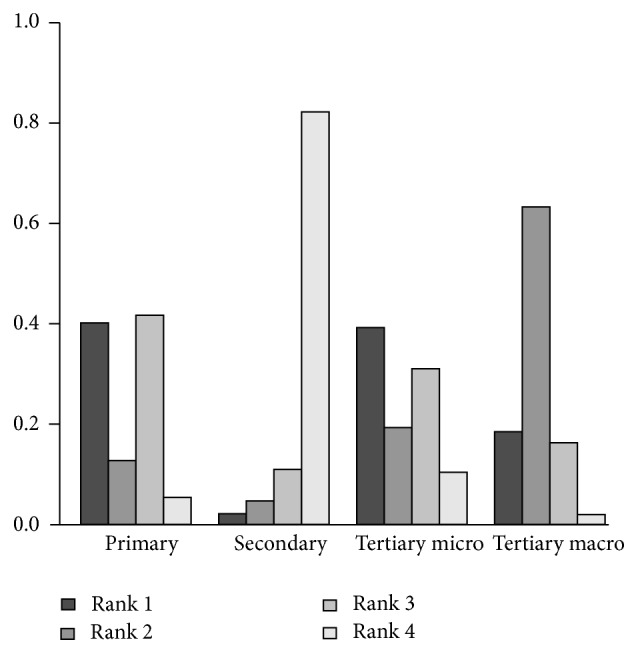
Rank acceptability indices for the base case scenario.

**Figure 4:**
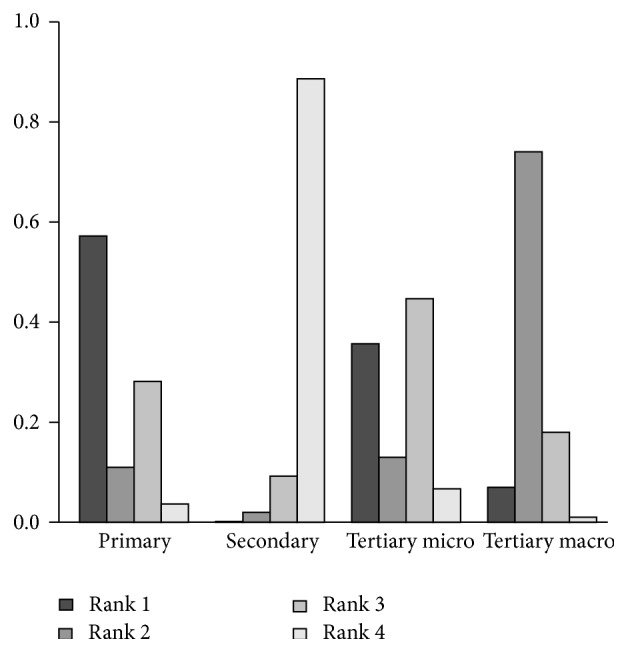
Rank acceptability indices when improvement of commercial headroom is favored.

**Figure 5:**
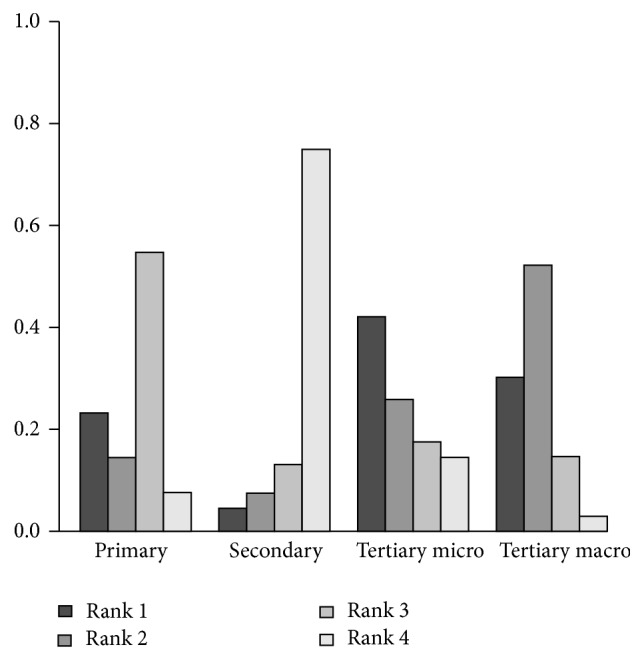
Rank acceptability indices when avoidance of barriers is favored.

For the preference-free analysis (Figure 3), we found that secondary prevention has a very low (<0.05) first rank acceptability index, making it unlikely to be optimal for any decision maker. The optimality of the three remaining strategies was however strongly dependent on the decision maker's preferences. Primary prevention was very likely to be the best alternative when maximizing the commercial headroom available is considered more important than minimizing the barriers and constraints to utilize this headroom (Figure 4). This is confirmed when looking at the pairwise winning indices, which show that the probability that primary prevention is preferred over tertiary prevention of microvascular complications, the second best alternative when improvement of commercial headroom is favored, is 61% (Table 2). Contrarily, tertiary prevention of microvascular complications and tertiary prevention of macrovascular complications were clearly the preferred strategies when having to deal with lesser obstacles is preferred over potential higher gains in terms of the objectives stated by the stakeholders (Figure 5). However, as is shown by the pairwise winning indices for this scenario (Table 3), the provided preference information with respect to the values of the weights was not precise enough to further discriminate between these two remaining strategies.

## 4. Discussion

Priority setting for translational research is a complex problem that requires decision makers to gather and synthesize expertise from different fields. In this paper, we have shown through a case study how this process can be supported in a formal way by applying MCDA.

The complete value chain in biomedical innovation poses a complex and multifaceted problem for priority setting. Additionally, ethics, public opinion, and politics come into play when dealing with a healthcare setting. Under these conditions, informal decision making will lead to the use of intuitive and heuristic approaches as a decision maker is unable to grasp the full complexity and trade-offs in a decision [17]. Informal decision making will therefore depend to a large extent on who is appointed to make the decision and what the background expertise of the decision maker (or group of decision makers) is, which would be undesirable in case of large investments or investments of public funds. The problem structuring phase of MCDA helps to overcome this by encouraging the incorporation of expertise exogenous to the decision makers. In our case study, this led to the integration of two different perspectives on the decision problem: that of the commercial headroom (based on the improvement in diagnostic power of new technologies over existing ones) and that of the barriers that new technologies would face to access the market. After the scoring phase it became apparent that the development of novel methods to measure biomarkers that can be used in secondary prevention of DM2 was certainly an unattractive research objective. If decision makers were willing to invest in all remaining three alternatives, the priority setting process could be stopped after this phase. However, in order to explore under which preferences the remaining alternatives would be most attractive, we proceeded with the preference modeling phase. A preference of decision makers for the maximization of commercial headroom made the development of novel methods to measure biomarkers used in primary prevention the most attractive strategy. Alternatively, investing in novel methods to measure biomarkers for tertiary prevention of microvascular and macrovascular complications was optimal in case a safer strategy with fewer obstacles, but less gain, would be preferred.

Early health economic modeling—the process of performing an initial assessment of the costs and health effects associated with a new medical technology before the technology has been fully developed—has recently been suggested as a tool to inform new product development within translational research projects [18–20]. However, given that such calculations require very strict assumptions about how a new technology performs in a specific clinical setting, this approach cannot yet be applied when specific biological targets still need to be identified. Other softer approaches such as SMAA-O are therefore required to support research priority setting at the start of a translational research project, where outcomes are generally too uncertain to make a full quantitative assessment of the expected return-on-investment meaningful. Using MCDA for priority setting at the beginning of a research project can facilitate decision making further on in the research and development process. For example, the data during the scoring phase can serve as input for quantitative approaches such as headroom analysis for product investment decision making [11] and value-based pricing for market access [21]. We therefore see SMAA-O or similar MCDA methods as a new instrument in the early health technology assessment toolbox, being one to be used at the very start of translational research projects.

A strength of the SMAA-O methodology that we employed in our case study is the possibility to combine ordinal and numerical scoring of the alternatives. This allowed us to make full use of the large amount of data available in the scientific literature on costs and health burden related to DM2, while still being able to incorporate expert judgment on aspects for which no data was available. A limitation of our study is that, apart from the scenarios considered, we did not elicit any preference information on the weights from the decision makers. Ordinal and ratio constraints on the weights can however easily be incorporated in a SMAA analysis by utilizing efficient weight generation techniques such as hit-and-run sampling [22].

We have demonstrated in this paper how the priority setting in translational research may be approached by applying MCDA. Future research is needed to fully assess the applicability of this method at the very start of a translational research project. Nonetheless, we are confident that we have already made a convincing case for formal decision making in priority setting in translational research. Our report may serve as a guide for future decision makers, ultimately making the approach common practice.

## Figures and Tables

**Table 1:** Scoring of the decision alternatives against the evaluation criteria.

	Preference direction	Primary prevention	Secondary prevention	Tertiary microvascular prevention	Tertiary macrovascular prevention
Reduction in downstream healthcare costs	Increasing	€ 658M	€ 0	€ 73M	€ 312M
Added quality-adjusted survival	Increasing	€ 280K	€ 0	€ 1K	€ 80K
Cost of related intervention	Decreasing	€ 792	€ 663	€ 155	€ 561
Feasibility of treat-all option		2	1	4	3
Performance of existing tests		3	4	1	2
Ease of implementation		2	2	1	1

**Table 2:** Pairwise winning indices when improvement of commercial headroom is favored.

	Primary prevention	Secondary prevention	Tertiary microvascular prevention	Tertiary macrovascular prevention
Primary prevention		0.96	0.61	0.65
Secondary prevention	0.04		0.07	0.02
Tertiary microvascular prevention	0.39	0.93		0.45
Tertiary macrovascular prevention	0.35	0.98	0.55	

**Table 3:** Pairwise winning indices when avoidance of barriers is favored.

	Primary prevention	Secondary prevention	Tertiary microvascular prevention	Tertiary macrovascular prevention
Primary prevention		0.88	0.35	0.31
Secondary prevention	0.12		0.18	0.12
Tertiary microvascular prevention	0.65	0.82		0.48
Tertiary macrovascular prevention	0.69	0.88	0.52	
